# Gastrointestinal Mucormycosis Mimicking Necrotizing Enterocolitis of Newborn

**Published:** 2013-10-01

**Authors:** V Raveenthiran

**Affiliations:** Department of Pediatric Surgery, Sri Ramasamy Memorial (SRM) Medical College, SRM University, Chennai 603203, India

 (Athena stands for abbreviation of Abstracting and Thoughtful Evaluation of Neonatal Articles; but it is also personified by the contributor. Like Athena of Greek mythology, she distills wisdom from published literature)

Like any other pediatric surgeon, Athena is often perplexed by the recalcitrant variety of necrotizing enterocolitis (NEC) in newborn. Despite appropriate therapy, they relentlessly progress to death. These cases are often shelved with a label “fulminant NEC”. [1] The mystery is slowly being unraveled by a recent series of articles on gastrointestinal mucormycosis of neonates (GIMN). GIMN masquerading as NEC is really an eye-opener.

The term ‘mucormycosis’ is not restricted to diseases caused by the genus Mucor; but is applicable to ailments caused by all the fungi of family Mucoraceae (order Mucorale). As Mucorale belongs to class Zygomycetes (under phylum Zygomycota), mucormycosis is also referred to, somewhat incorrectly, as zygomycosis. [2,3,4] They are ubiquitous saprophytic moulds that can cause invasive infection in immunocompromised individuals. Preterm neonates with immature immune system are more vulnerable for GIMN. In fact 83% of GIMN occur in preterm neonates. [5] Corticosteroid therapy of preterm neonates, [6] co-existing congenital heart disease with splanchnic hypoperfusion, [7] acidosis, [8] use (abuse) of broad spectrum antibiotics, [8] low gastric pH of 6.5 [9] and depriving of immunogenic breast-milk are additional factors that facilitate fungal invasion of neonatal gut. [7] 

Members of Mucoraceae are unique in their affinity for artery. [6] Arterial wall, which is usually resistant for microbial invasion, is easily penetrated by these fungi. The resultant arterial thrombosis cause extensive ischemic necrosis of the tissues supplied by the artery. Thus, the damage is often disproportionate to the degree of infection. [8] Intestinal gangrene caused by invading Mucorales mimics NEC. 

Neonates can acquire gastrointestinal infection by inhaling or ingesting fungal spores from air or contaminated hospital equipments. Warm humid environment of neonatal intensive care units are said to be ideal for fungal sprouting. [10] There are instances wherein GIMN was acquired from contaminated enteral feeds, [11] parenteral infusions, [12] nasogastric tubes, [13] endotracheal tubes [13] and wooden tongue depressors. [14] Report of a newborn, who presented with simultaneous gangrene of tongue and jejunum, confirms the trans-oronasal route of Mucorale infection. [9]

The opportunistic pathogens of the family Mucoraceae include the genera Mucor, Rhizopus, Absidia and Rhizomucor. [6] Absidia infection of neonatal gut has been reported only twice. [3, 15] Although fewer than 25 cases of GIMN have been reported so far, exact species-identification has not been documented in most of the papers. The reason for this is the retrospective nature of diagnosis in more than 75% of patinets. [5] In some cases, the correct diagnosis is made several months after discharge or death of the neonate. [16] Antemartem and preoperative diagnoses are exceptions rather than the rule. [17] The clinical features of GIMN are often mistaken for NEC or spontaneous perforation of Hirschsprung disease. [8, 18, 19, 20] Abdominal distension, bilious vomiting, lethargy and thrombocytopenia are common to all the three conditions. 

The clinical clue to differential diagnosis lies in the nosopoietic mechanism of NEC and GIMN. (Table 1) As NEC is essentially a reperfusion injury, the affected bowel will be congested, moist and it will undergo putrefaction. On the other hand, as GIMN is due to thrombotic occlusion of mesenteric artery, the affected gut will be avascular, dry and it will undergo anemic-infarction. Therefore, contrary to NEC, rectal bleeding, pneumatosis intestinalis and portal vein gas are unusual in GIMN. [6, 21] Ischemic bowel of GIMN, in an attempt to imbibe oxygen, adheres with adjacent healthy bowel. This explains the early occurrence of palpable masses [4, 22] and rarity of free perforations (pneumoperitoneum) [9] in GIMN. Palpable abdominal mass was noted at presentation in 16% of GIMN. [5] The mass can be as big as 10 x 10 cm and hence mistaken for a solid tumor (neuroblastoma). [22]

**Figure:**
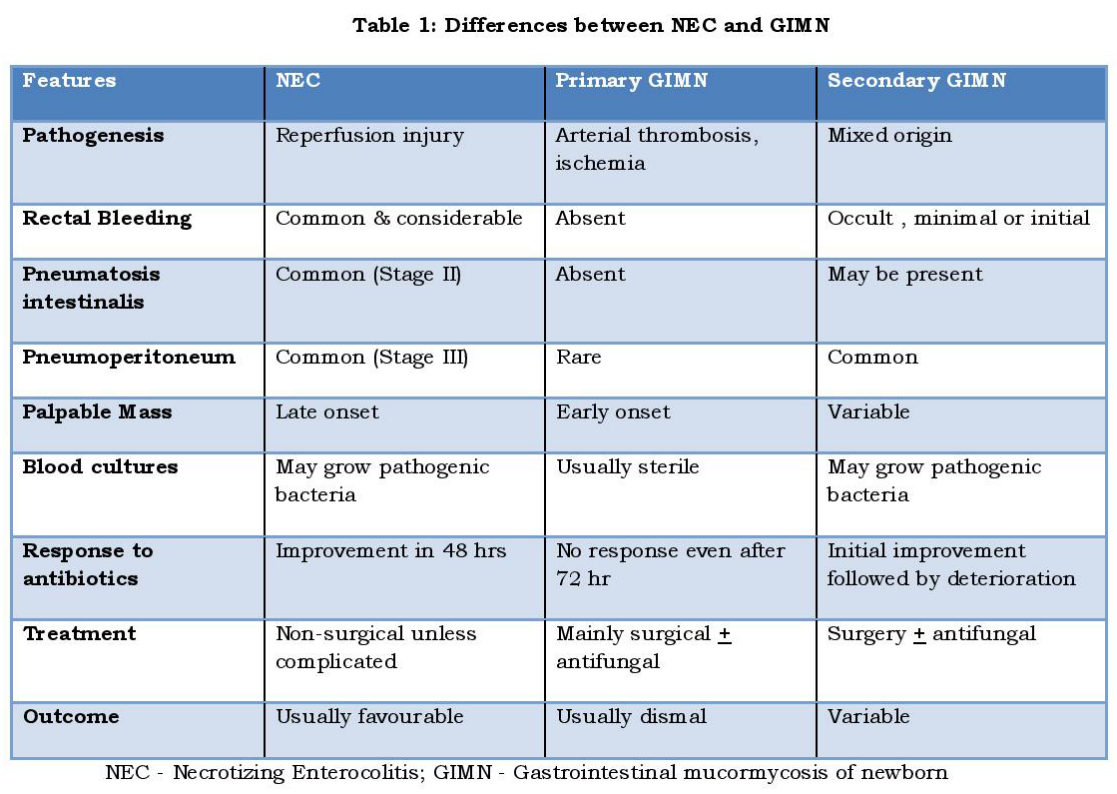
Table 1

Contrary to the foregoing descriptions, Athena is aware of reports describing rectal bleed, [8, 23] pneumoperitoneum [8, 24] and pneumatosis intestinalis [7, 25] in GIMN. Kargl et.al [7] suggested that a thickened bowel in ultrasonography is suggestive of GIMN; but Mohta et.al [23] noted thinning of the intestinal wall during surgery. Based on these paradoxical presentations, Athena believes that there are two forms of GIMN. (Table 1) In primary GIMN, invasive infection occurs when the fungal colonization of neonatal gut exceeds a critical size. This form appears to be extremely rare. In secondary GIMN the fungal infection itself is secondary to the predisposing NEC. Devitalized tissue of NEC provides a good medium for the saprophytic fungi to grow. In secondary GIMN, clinical presentation will, as expectedly, have mixed features of both NEC and GIMN. 

It is essential to distinguish secondary GIMN from simple fungal colonization or contamination. [26] This is done only by histological demonstration of fungal hyphae in viable tissue using special stains such as Periodic Acid Schiff (PAS) or Gomori silver methnamine. [8] Importantly, the empty-looking ribbon-like fungal hyphae do not stain with Hematoxylin-Eosin and hence the diagnosis is likely to be missed by routine histological processing. [3] Fungal cultures are not routinely done because they do not distinguish invasive infection from innocuous colonization. Further fungal cultures are time consuming and are positive only in 33% cases. [9] For these reasons many authors emphasize on histopathology rather than mycological cultures for diagnosing GIMN. [8, 26] But Athena considers supplementary fungal culture (of necrotic tissue) essential for species identification and susceptibility testing. Although positive stool cultures are not diagnostic, they may raise the suspicion of GIMN. As histopathology and culture may take several days for reporting, Patra et.al [8] recommended frozen section in addition to the routine paraffin preparations. 

The role of antifungal drugs in the treatment of GIMN is contentious. Liposomal Amphotericin B and Fluconazole have been used in many cases. [13] But, interesting are the reports wherein neonates recovered without any specific antifungal therapy. [16, 24] For example, in Nichol’s case the diagnosis was established only 3 months after the patient was discharged from hospital. [16] Mycotic thrombus of feeding arteries is thought to prevent effective distribution of systemic antifungal drugs into the infected tissue. [3, 27] Aggressive and wide surgical debridement is acknowledged as the mainstay in treatment of GIMN. 

Following the resection of ischemic intestines, primary anastomosis is obviously undesirable. Sarin [13] performed decompressing enterostomy and successfully restored it without any complications. But, Athena would learn lessons from the unfortunate case of Inoue et.al. [4] The neonate developed extensive gangrene of anterior abdominal wall, probably due to fungal invasion from the enterostomy edges. Athena consider it prudent to “clip and drop” the resected edges. Abdominal incision is best left as laparostomy, enabling periodic inspection of resected ends. Delayed primary anastomosis can be done when the resected ends are histologically proven negative for fungal hyphae. [28] 

Mortality of GIMN is as high as 75%. [6, 8, 29] This high mortality is partly due to delay in diagnosis and partly due to inappropriate or inadequate treatment. [28, 29] Finally, Athena is fascinated by three things: (i) Nearly 70% of the reported cases of GIMN are from India. (ii) GIMN has a peculiar predilection for colon [30] and (iii) GIMN has been reported as early as 1959 [32] and it took another 4 decades to rediscover it. [21, 33]

## Footnotes

**Source of Support:** Nil

**Conflict of Interest:** The author is Editor of the journal. But he did not take part in the evaluation or decision making of this manuscript. The manuscript has been independently handled by two other editors.

